# Comparative Transcriptome Analysis of Differentially Expressed Genes and Signaling Pathways between XY and YY Testis in Yellow Catfish

**DOI:** 10.1371/journal.pone.0134626

**Published:** 2015-08-04

**Authors:** Junjie Wu, Shuting Xiong, Jing Jing, Xin Chen, Weimin Wang, Jian-Fang Gui, Jie Mei

**Affiliations:** 1 College of Fisheries, Key Laboratory of Freshwater Animal Breeding, Ministry of Agriculture, Freshwater Aquaculture Collaborative Innovation Center of Hubei Province, Huazhong Agricultural University, Wuhan, 430070, China; 2 State Key Laboratory of Freshwater Ecology and Biotechnology, Institute of Hydrobiology, Chinese Academy of Sciences, University of the Chinese Academy of Sciences, Wuhan, 430072, China; The Ohio State University, UNITED STATES

## Abstract

YY super-males have rarely been detected in nature and only been artificially created in some fish species including tilapia and yellow catfish (*Pelteobagrusfulvidraco*), which provides a promising model for testis development and spermatogenesis. In our previous study, significant differences in morphology and miRNA expression were detected between XY and YY testis of yellow catfish. Here, solexa sequencing technology was further performed to compare mRNA expression between XY and YY testis. Compared with unigenes expressed in XY testis, 1146 and 1235 unigenes have significantly higher and lower expression in YY testis, respectively. 605 differentially expressed unigenes were annotated to 1604 GO terms with 319 and 286 genes having relative higher expression in XY and YY testis. KEGG analysis suggested different levels of PI3K-AKT and G protein-coupled receptor (GPCR) signaling pathways between XY and YY testis. Down-regulation of miR-141/429 in YY testis was speculated to promote testis development and maturation, and several factors in PI3K-AKT and GPCR signaling pathways were found as predicted targets of miR-141/429, several of which were confirmed by dual-luciferase reporter assays. Our study provides a comparative transcriptome analysis between XY and YY testis, and reveals interactions between miRNAs and their target genes that are possibly involved in regulating testis development and spermatogenesis.

## Introduction

Sex determination and differentiation are the most significant developmental events that direct the embryonic gonads into either testes or ovaries [1,2]. The XY sex-determining system is the most popular sex-determination system found in vertebrates. Sry and dmrt1 are conserved sex-determining genes, triggering differentiation of testes from bi-potential gonads in mammals and birds [3,4]. Until now, multiple sex-determining genes including *dmy/dmrt1Y* and *dmrt1* in *Oryzias latipes* [5,6], *gsdf* in *Oryzias luzonensis* [7], *sox3* in *Oryzias dancena* [8], *amhy* in *Odontesthes hatchery* [9], *sdY* in *Oncorhynchus mykiss* [10], *amhr2* in *Takifugu rubripes* [11] and *dmrt1* in *Cynoglossus semilaevis* [12] have been identified to participate in the male sex determination in different fish species. Moreover, several important genes such as *cyp19a1*, *foxl2*, *wnt4* for ovary differentiation and *dmrt1*, *sox9*, *amh* for testicular differentiation have been revealed in teleosts [13–15].

As an evolutionary link between invertebrates and higher vertebrates, fish species have a very complex sex determination system with XX/XY male heterogametic system as the main sex determination system [16]. Until now, YY super-males have only been artificially produced in some fish species [17–19], but they could not survive in case of higher vertebrates including human and mouse. Previous studies on rainbow trout (*Oncorhynchusmykiss*) reported obvious differences in gene expression and morphology between XY and YY testis [20,21]. Significant differences of aromatase expression were found in spermatogonia, spermatids and epithelial cells among XY and YY testis [21]. Moreover, a relative higher level of androgen receptor expression was observed in efferent ducts of YY testis compared with XY testis [20]. However, the sperm quality and quantity of XY and YY males were the similar in Nile tilapia (*Oreochromisniloticus*)[22]. There are no comprehensive studies regarding genetic differences between XY and YY males.

In our previous study, we observed larger spermatogenic cyst and more spermatids in YY super-males than XY males by histological analysis, suggesting a higher degree of testis maturity. Many miRNAs that are potentially involved in testis development and spermatogenesis were identified to be differentially expressed between XY and YY testis [23]. The establishment and maintenance of spermatogenesis in fish requires specialized gene regulatory networks in the testis [24]. Here, we utilized RNA-Seq approach to identify genes and pathways that were differentially expressed between XY and YY testis and their functional relationship with miRNAs. Hopefully, our findings would provide a clue about the genetic mechanism of testicular differences between XY and YY males.

## Results

### Illumina sequencing, sequence assembly and functional annotation

In order to identify differentially expressed mRNA in testes of male and super-male yellow catfish, two solexa libraries were constructed by XY and YY testes respectively. After removing adaptors and low quality reads, a total of 87,149,414 and 75,448,188 reads were obtained in each profile respectively. After *de novo* assembly, 78148 unigenes were obtained by paired-end method of Trinity and TGICL clustering with mean length of 944bps (S1 Fig).

**Fig 1:**
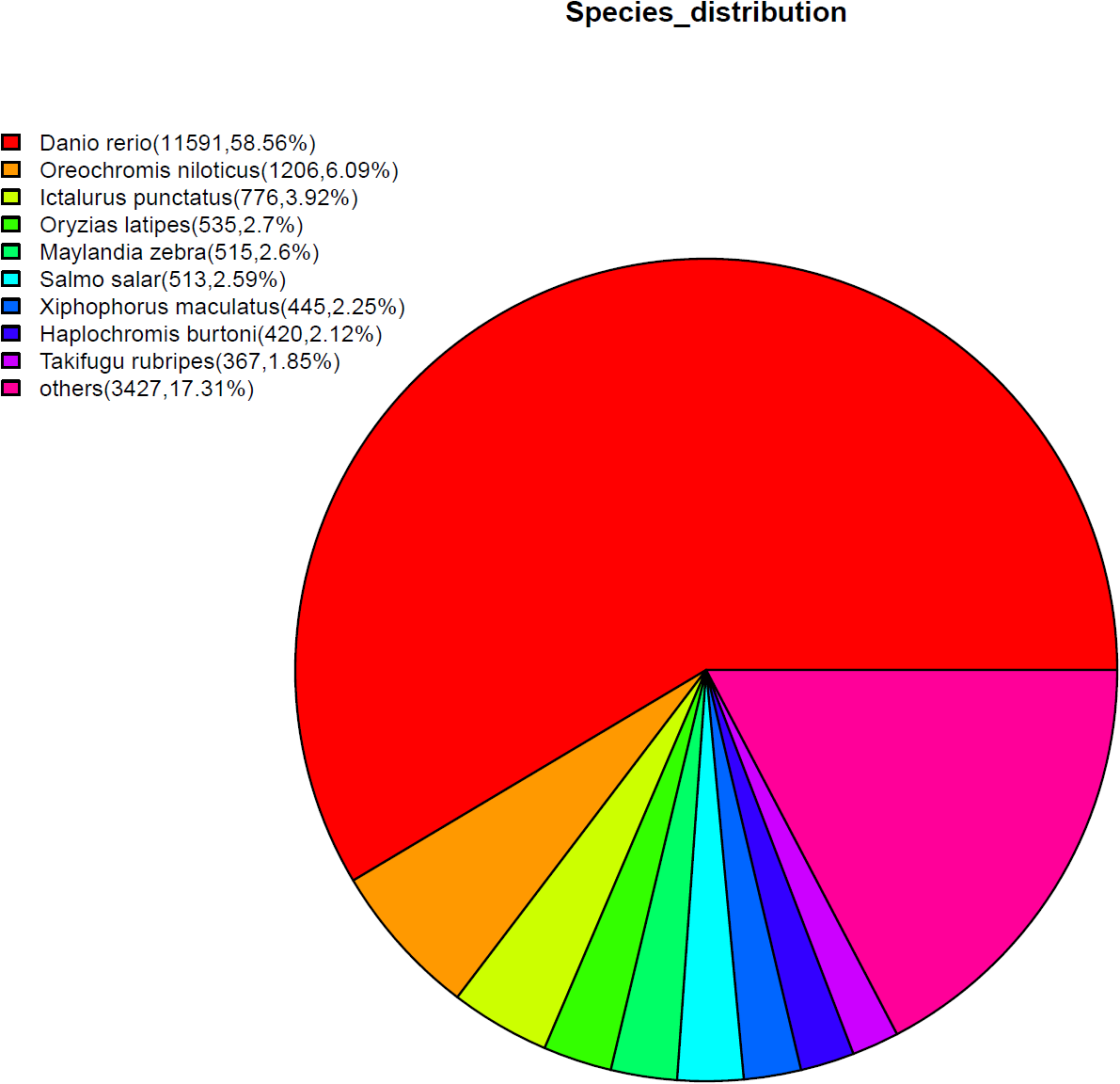
Species distribution of the top BlastX hits using the assembled unigenes of yellow catfish. The unigenes were searched against the NCBI Nr database with a cut-off E-value<1e-5.

The unigenes of *de novo* assembly were searched against the NCBI non-redundant (nr), SWISS-PROT, KEGG, GO and KOG protein databases by using BLASTX with a cut-off E-value of 1e^-5^ (S1 Table). Finally, a total of 19,795 (25.33%) unigenes were significantly matched with nr database. Among these BlastX-hit unigenes (Fig 1), 11591 (58.56%) hits were assigned to *Daniorerio* while only 776 (3.92%) hits overlapped with *Ictalurus punctatus* (the close species to *Pelteobagrus fulvidraco*), perhaps due to the limited amount of genomic data on GeneBank about the species of Siluriformes. In addition, there were a number of unigenes without hitting results, which may contain non-coding fragments or some undetected genes.

### Analysis of differentially expressed genes (DEGs)

**Fig 2:**
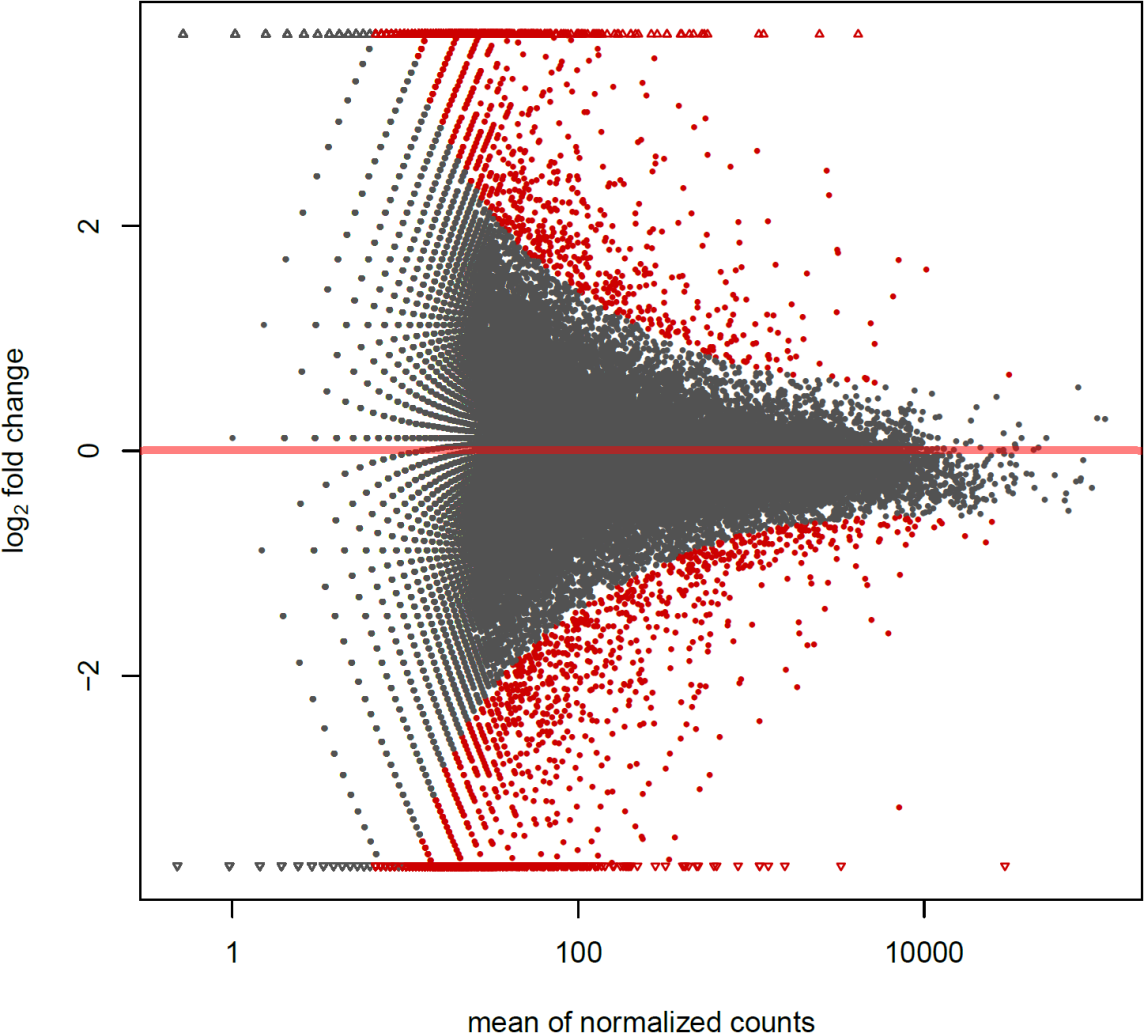
Difference of unigene expression between male (XY) and super-male (YY). The X-axis represents the value of normalized expression counts, and the Y-axis represents the level of differential expression. Significantly differential expressions are marked in red and other in grey.

To identity differentially expressed unigenes between XY male and YY super-male, the expression of assembled unigenes were counted by RPKM method. After comparison (XY*vs*YY testis) with fold change threshold value = 2 and FDR test (*P*< 0.05), 4458 unigenes were found expressed with significant difference. Among them 1006 were detected from testis of YY super-male and 1072 were found from testis of XY male. Besides this, 1146 unigenes from YY and 1235 from XY testis were detected with significantly higher expression. The MA scatter plots comparison reveal the differential expressed genes existed between testes of male and super-male in yellow catfish (Fig 2).

### GO annotation and analysis of DEGs enriched in YY testis

**Fig 3:**
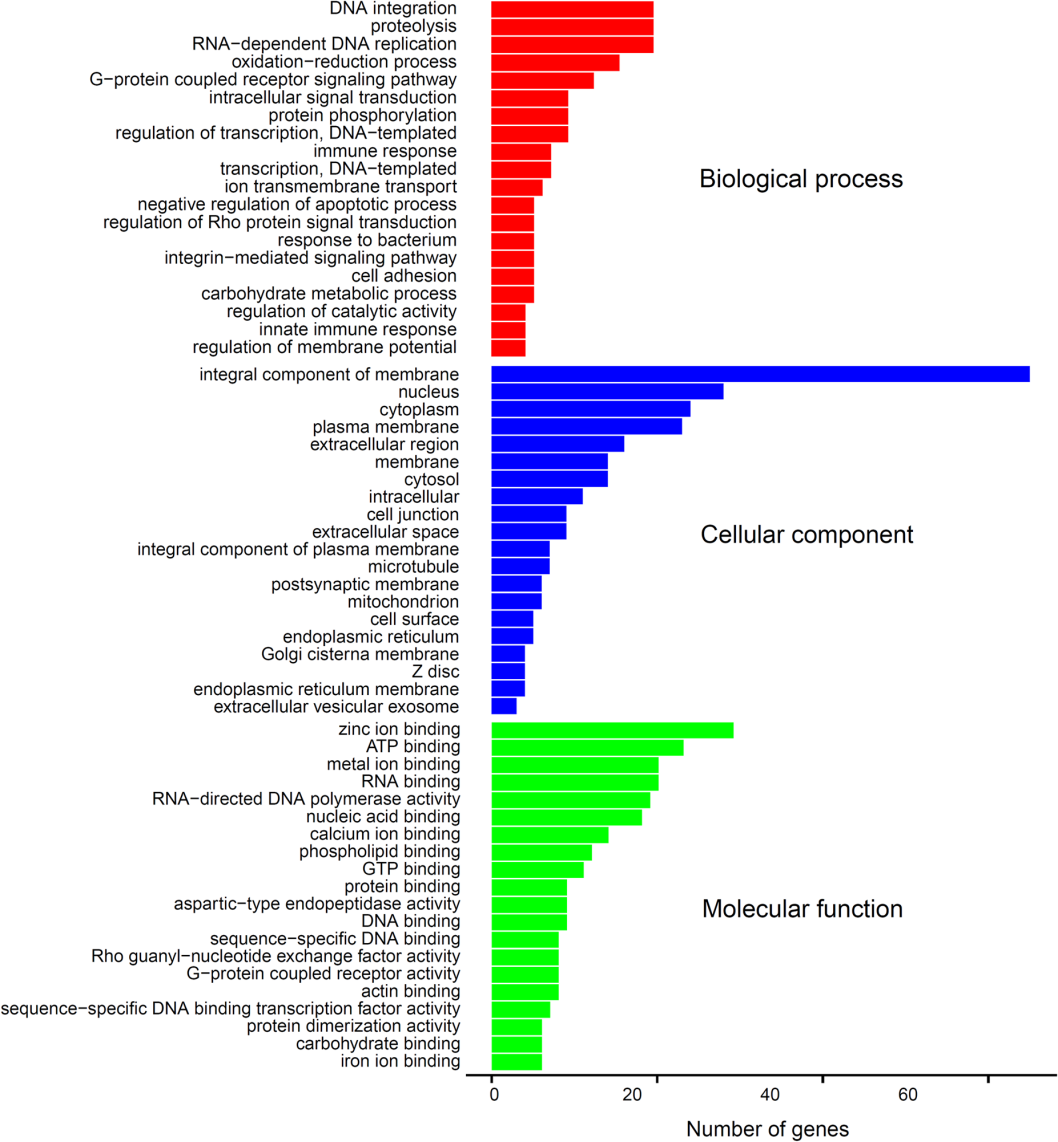
Functional classification of the DEGs that has higher expression in YY than XY based on gene ontology (GO) terms: biological process (red), cellular component (blue),molecular function (green). The X-axis is number of unigenes.

The 4458 differentially expressed unigenes (DEGs) were searched against the Gene Ontology database (www.geneontology.org) to determine which kinds of GO term DEGs mainly participated in. Finally, 605 DEGs were annotated to 1604 GO terms (S2 Table) accompanied by 319 and 286 genes with relative higher expression in XY and YY testes, respectively. The functions of 286 DEGs with higher expression in YY than XY testis were assigned to biological process, cellular component and molecular function (Fig 3). In biological process, proteolysis (58 DEGs, GO:0006508), RNA-dependent DNA replication (38 DEGs, GO: 0006278) and DNA integration (33DEGs, GO:0015074) were the most prominent terms. Integral component of membrane (112 DEGs,GO:0016021) was the most prominent within cellular component followed by nucleus (67DEGs,GO:0005634), cytoplasm (48DEGs,GO:0005737) and plasma membrane (40DEGs,GO:0005886). In molecular function, most of the annotated unique sequences were assigned to zinc ion binding (74DEGs,GO:0008270), ATP binding (51DEGs,GO: 0005524), metal ion binding (53DEGs,GO:0046872), RNA binding (46 sequences,GO:0003723) and RNA-directed DNA polymerase activity (38DEGs,GO:0003964)(Fig 3).

### KEGG analysis of DEGs between XY and YY testis

**Fig 4:**
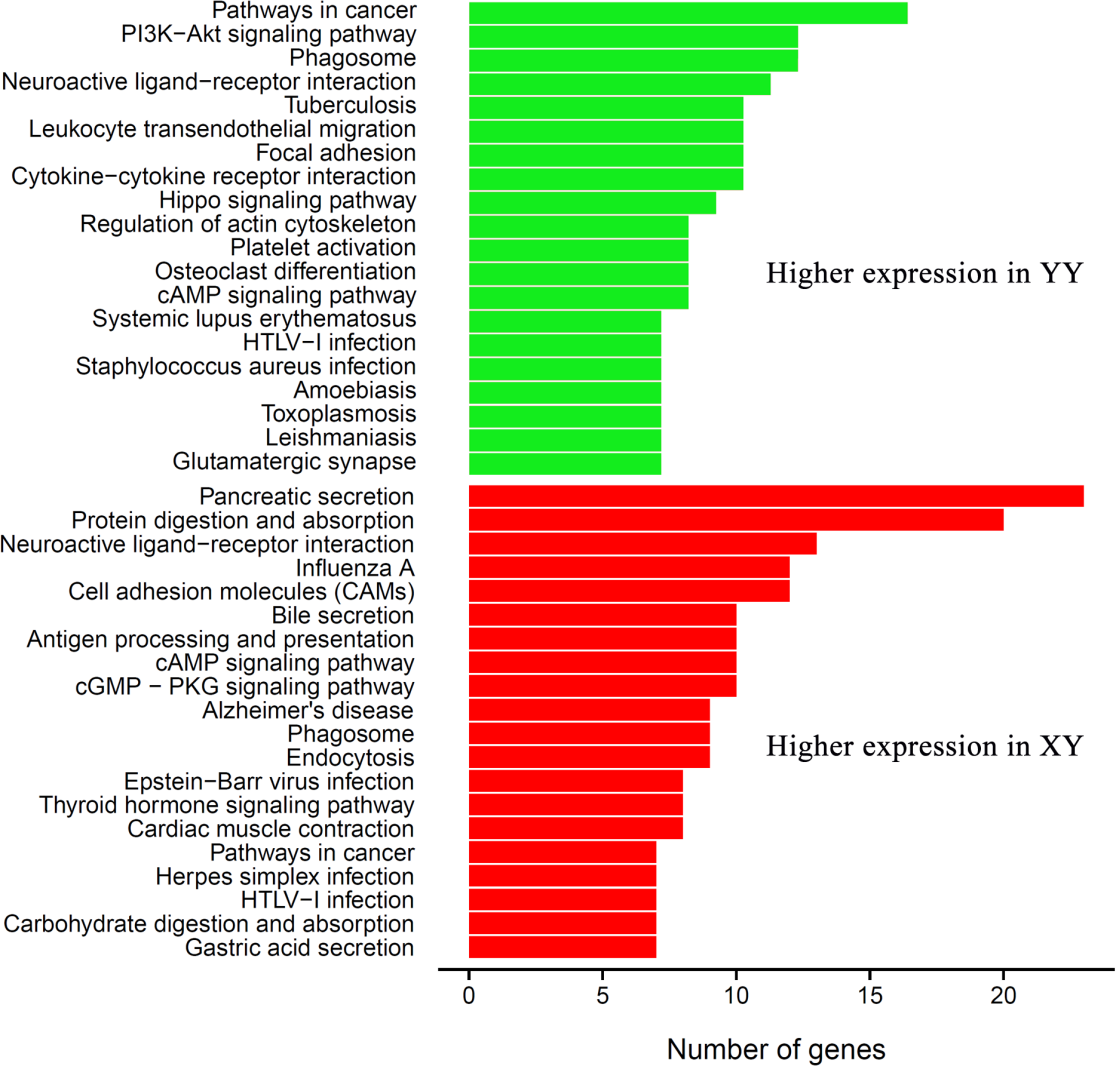
KEGG classification of the DEGs between transcriptomes of YY and XY testis. The green and red columns represent the signaling pathways enriched in YY and XY testes, respectively.

KEGG annotation is the process of mapping interested genes to the metabolic pathways. In our study, 312 DEGs were mapped to 252 KEGG pathways. Enrichment analysis shows that 143 YY highly expressed unigenes were enriched in 192 pathways, and most of the DEGs were assigned to pathways in cancer (16 DEGs, ko05200), PI3K-Akt signaling pathway (12 DEGs, ko04151), phagosome (12 DEGs, ko04145), neuroactive ligand-receptor interaction (11 DEGs, ko04080), cytokine-cytokine receptor interaction (10 DEGs, ko046760), tuberculosis (10 DEGs, ko05152) and focal adhesion (10 DEGs, ko4510). The XY highly expressed unigenes were enriched in pancreatic secretion (23 DEGs, ko04972), protein digestion and absorption (20 DEGs, ko04974) and neuroactive ligand-receptor interaction (13 DEGs, ko04080) (Fig 4).

**Fig 5:**
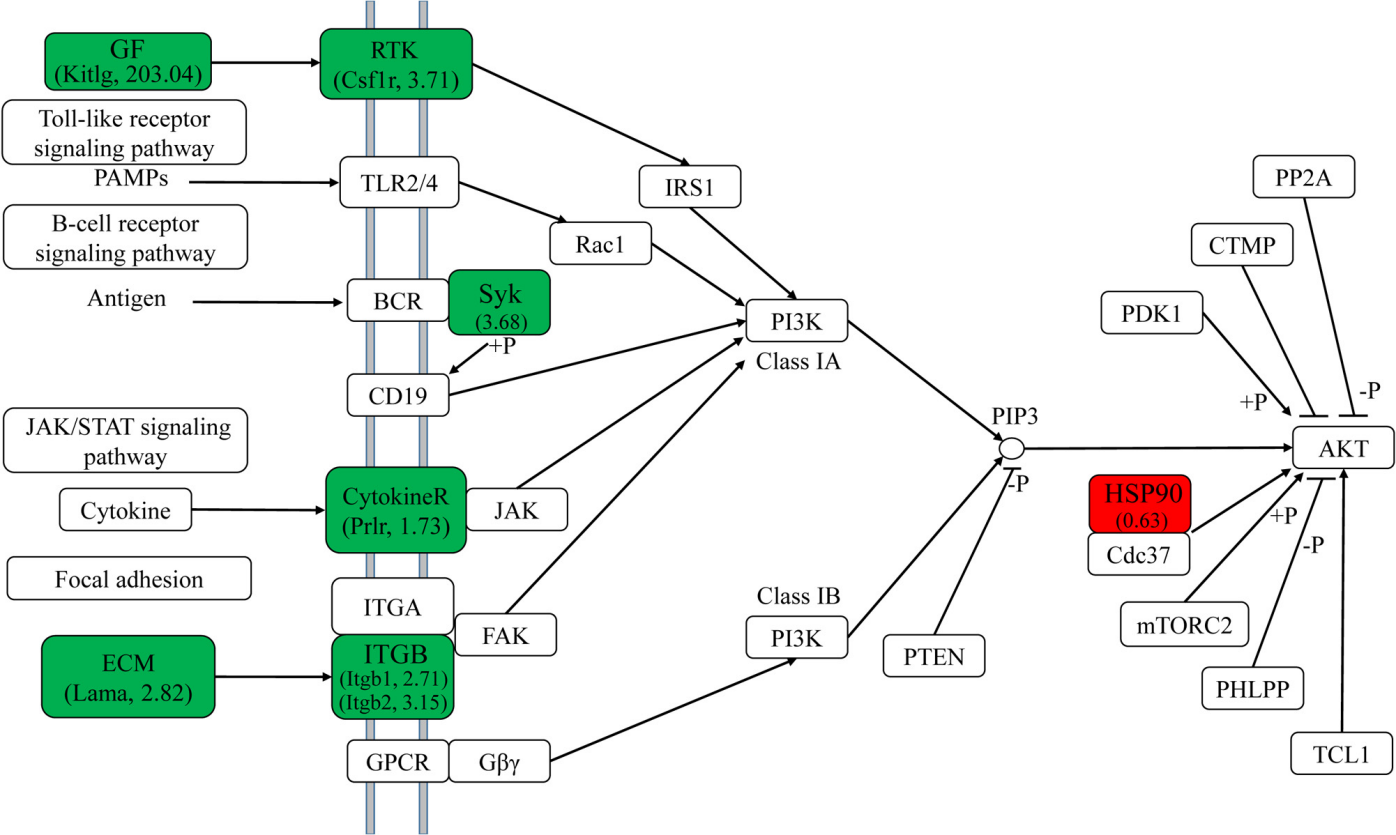
DEGs involved in the PI3K-AKT signaling pathway. YY highly expressed unigenes are shown in green and XY highly expressed unigenes are shown in red. The numbers in parentheses indicate the value of fold-change (RPKM value of XY/ RPKM value of YY).

**Fig 6:**
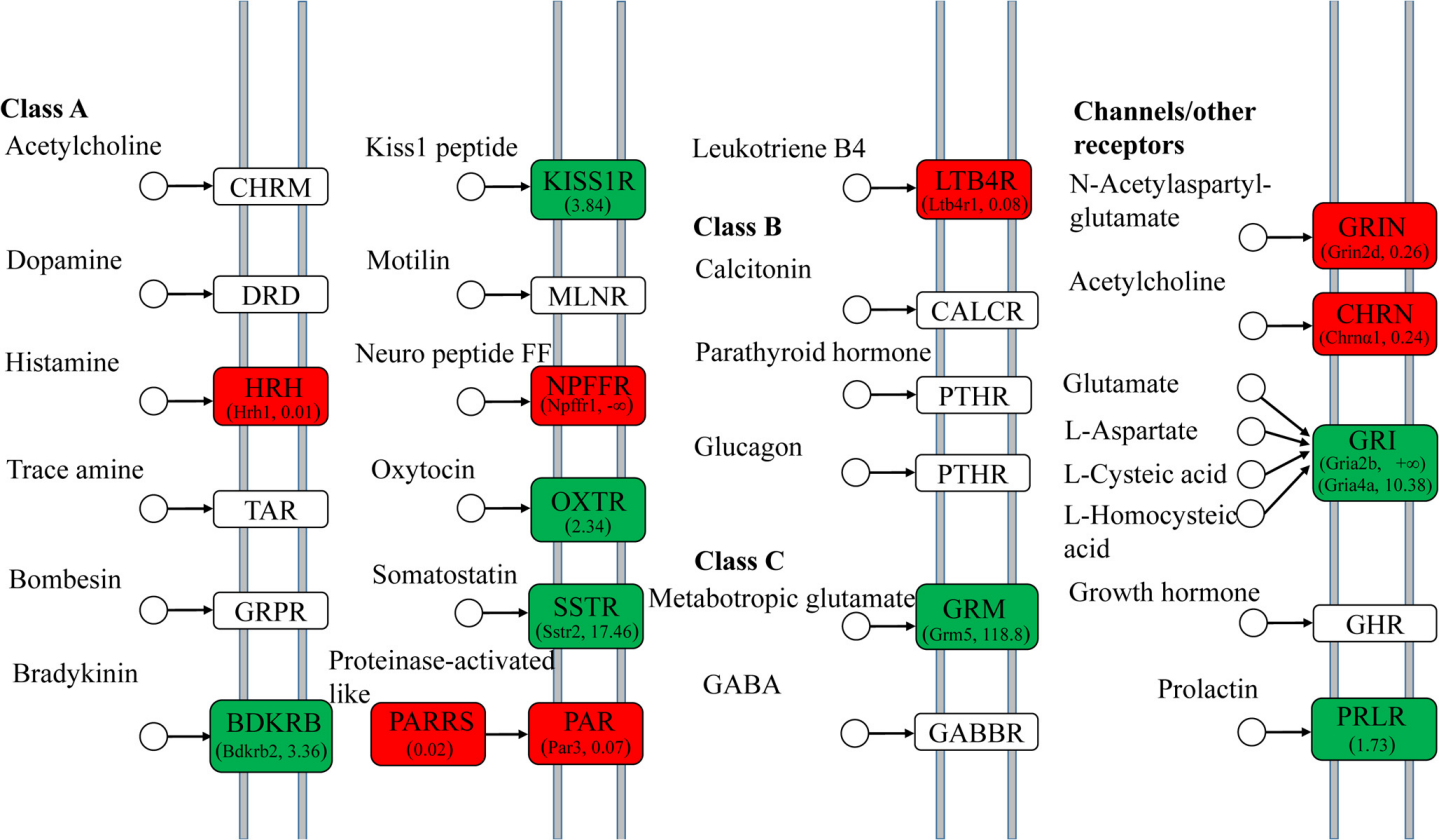
DEGs involved in the GPCR signaling pathway. YY highly expressed unigenes are shown in green and XY highly expressed unigenes are shown in red. The numbers in parentheses indicate the value of fold-change (RPKM value of XY/ RPKM value of YY).

The PI3K-AKT signaling pathway is involved in many fundamental functions including testis development and spermatogenesis, and stimulated by many kind of growth factors that specifically binds to receptor tyrosine kinase (RTK) or G protein-coupled receptors (GPCR) [25,26]. As members of RTK, the spleen tyrosine kinase (Syk), colony stimulating factor 1 receptor (Csf1r), and prolactin receptor (Prlr) were expressed about 3.68, 3.71 and 1.73 fold higher in YY testis than in XY, respectively. In addition, β1-Integrin (Itgb1) and β2-Integrin (Itgb2) were also expressed 2.71 and 3.15-fold higher in YY testis than in XY (Fig 5). In the “neuroactive ligand–receptor interaction” signaling pathway, expression of multiple genes associated with G protein signaling were significantly up-regulated in YY testis (Fig 6), like Kiss1r (GPR54) and somatostatin receptors (Sstr) in Class A of GPCR signaling and metabotropic glutamate receptor 5 (GRM5) in Class C of GPCR signaling. Meanwhile, the mRNA levels of glutamate receptor AMPA 2b (gria2b), glutamate receptor AMPA 4a (gria4a) and prolactin receptor (PRLR) were also higher in YY than in XY. In contrast, the expression levels of histamine receptor H1 (Hrh1), neuropeptide FF receptor 2 (Npffr2), leukotriene B4 receptor 1 (Ltb4r1) and nicotinic acetylcholine receptor α1 (Chrnα1) were higher in XY than in YY testis.

### qRT-PCR confirmation of DEGs between XY and YY testis

**Fig 7:**
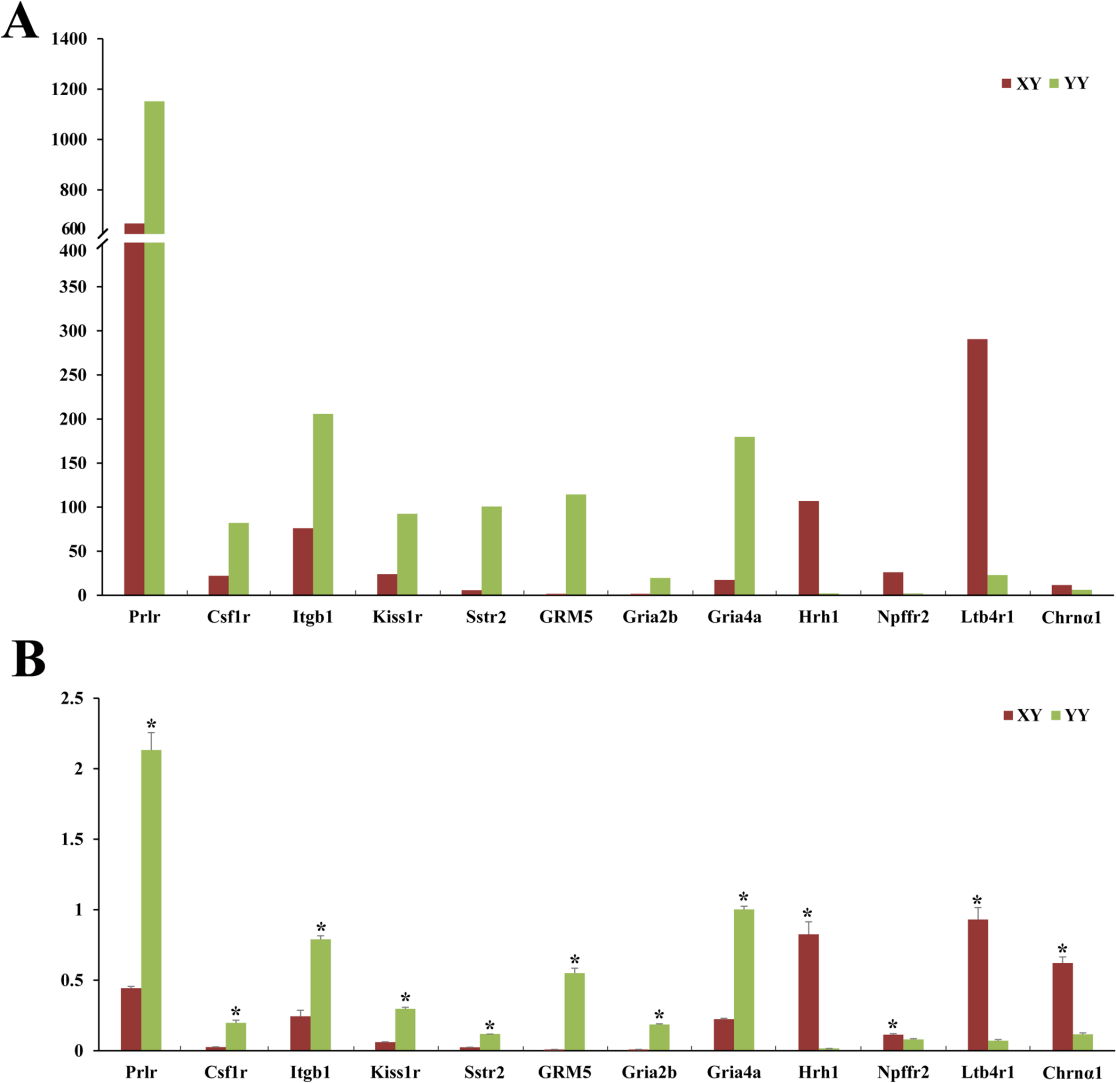
Concordance of solexa sequencing data with qRT-PCR data. (A) Profile of solexa sequencing value for selected genes with normalized expression data. (B) Profile of relative expression of selected genes by qRT-PCR. *p<0.05 indicates the significant difference in gene expression between male (XY) and super-male (YY).

To verify the accuracy of the sequencing data, twelve DEGs related to RTK and G protein signaling pathway were arbitrarily selected and validated by quantitative real-Time PCR (qRT-PCR). The twelve DEGs includes four genes (Hrh1, Npffr2, Ltb4r1, Chrnα1) relatively high expressed in XY and eight genes (Prlr, Csf1r, Itgb1, Kiss1r, Sstr, GRM5, Gria2b, Gria4a) relatively high expressed in YY testis in the transcriptome data (Fig 7A). As in the qRT-PCR results, the relative expression levels of twelve differentially expressed genes were completely consistent with the Solexa sequencing (Fig 7B).

### Identification of potential target genes for miR-141/429 from DEGs and validation by dual-luciferase reporter assays

In our previous study, relative lower expression of miR-141/429 was observed in YY testis indicating a higher degree of testis maturity than XY testis. A high dose of 17α-ethinylestradiol (EE2) up-regulates the expression of miR-141/429 [23]. Here, we examined whether some DEGs are potential target genes for miR-141-3p and miR-429b-3p. Finally, 31 and 11 YY enriched DEGs were predicted to be targeted by miR-141-3p and miR-429b-3p (Table 1). For example, Itgb2, a factor involved in the PI3K-AKT signaling pathway, was highly expressed in YY and was a putative target of miR-141-3p. In addition, Gria4a is a factor for the neuroactive ligand–receptor interaction signaling pathway and also a putative target for miR-141-3p. AMH and Tgfβr1 were potential targets for miR-141-3p and miR-429b-3p, respectively.

**Table 1:** The predicted targets of miR141/429 that displayed higher expression in YY than XY.

miRNA name	No. of tanscript	Putative target	Target gene annotation
pfu-miR-141-3p	CL52176Contig1	Aacs	acetoacetyl-CoA synthetase
pfu-miR-141-3p	CL51317Contig1	Amh	anti-mullerian hormone
pfu-miR-141-3p	CL40242Contig1	Amph	amphiphysin
pfu-miR-141-3p	CL55509Contig1	Bcl6	B-cell lymphoma 6 protein
pfu-miR-141-3p	CL12469Contig1	Cacnα2d4	voltage-dependent calcium channel alpha-2/delta-4
pfu-miR-141-3p	CL75180Contig1	Chrd	chordin
pfu-miR-141-3p	CL9871Contig1	Col6α	collagen type VI alpha
pfu-miR-141-3p	CL1267Contig1	Ctnna	catenin alpha
pfu-miR-141-3p	CL74732Contig1	Cxcl13	C-X-C motif chemokine 13
pfu-miR-141-3p	CL52163Contig1	Dmbt1	deleted in malignant brain tumors 1 protein
pfu-miR-141-3p	comp12586_c0_seq1	Dnah	dynein heavy chain, axonemal
pfu-miR-141-3p	CL54671Contig1	Nsdhl	sterol-4alpha-carboxylate 3-dehydrogenase (decarboxylating)
pfu-miR-141-3p	CL15376Contig1	Ext1	glucuronyl/N-acetylglucosaminyl transferase EXT1
pfu-miR-141-3p	CL49906Contig1	Fads2	fatty acid desaturase 2 (delta-6 desaturase)
pfu-miR-141-3p	CL53372Contig1	Gli2	zinc finger protein GLI2
pfu-miR-141-3p	CL72487Contig1	Gria4	glutamate receptor 4
pfu-miR-141-3p	CL15787Contig1	Hya	hyaluronoglucosaminidase
pfu-miR-141-3p	CL53574Contig1	Itgb2	integrin beta 2
pfu-miR-141-3p	CL14671Contig1	Lamα4	laminin alpha 4
pfu-miR-141-3p	CL4177Contig1	Ldlrap1	low density lipoprotein receptor adapter protein 1
pfu-miR-141-3p	CL29924Contig1	Mep1b	meprin B
pfu-miR-141-3p	CL56196Contig1	Mx1	interferon-induced GTP-binding protein Mx1
pfu-miR-141-3p	CL7053Contig1	Myh	myosin heavy chain
pfu-miR-141-3p	CL54823Contig1	Nadph	hydroxymethylglutaryl-CoA reductase (NADPH)
pfu-miR-141-3p	CL54540Contig1	Nod1	nucleotide-binding oligomerization domain-containing protein 1
pfu-miR-141-3p	CL27176Contig1	Ntrk3	neurotrophic tyrosine kinase, receptor type 3
pfu-miR-141-3p	CL53494Contig1	Oxtr	oxytocin receptor
pfu-miR-141-3p	CL37449Contig1	Prlr	prolactin receptor
pfu-miR-141-3p	CL5783Contig1	Sema3	semaphorin 3
pfu-miR-141-3p	CL13367Contig1	Socs3	suppressor of cytokine signaling 3
pfu-miR-141-3p	CL47996Contig1	Ttn	titin
pfu-miR-429b-3p	CL54671Contig1	Erg26	sterol-4alpha-carboxylate 3-dehydrogenase (decarboxylating)
pfu-miR-429b-3p	CL15376Contig1	Ext1	glucuronyl/N-acetylglucosaminyl transferase EXT1
pfu-miR-429b-3p	CL49906Contig1	Fads2	fatty acid desaturase 2 (delta-6 desaturase)
pfu-miR-429b-3p	CL55088Contig1	Fmn2	formin 2
pfu-miR-429b-3p	CL15787Contig1	Hya	hyaluronoglucosaminidase
pfu-miR-429b-3p	CL53522Contig1	Lamα1	laminin, alpha 1
pfu-miR-429b-3p	CL2394Contig1	Lamα3	laminin, alpha 3
pfu-miR-429b-3p	CL55132Contig1	Mmp9	matrix metalloproteinase-9 (gelatinase B)
pfu-miR-429b-3p	CL61104Contig1	Mylk	myosin-light-chain kinase
pfu-miR-429b-3p	CL15079Contig1	Sec61α	protein transport protein SEC61 subunit alpha
pfu-miR-429b-3p	comp105559_c1_seq2	Tgfβr1	TGF-beta receptor type-1

**Fig 8:**
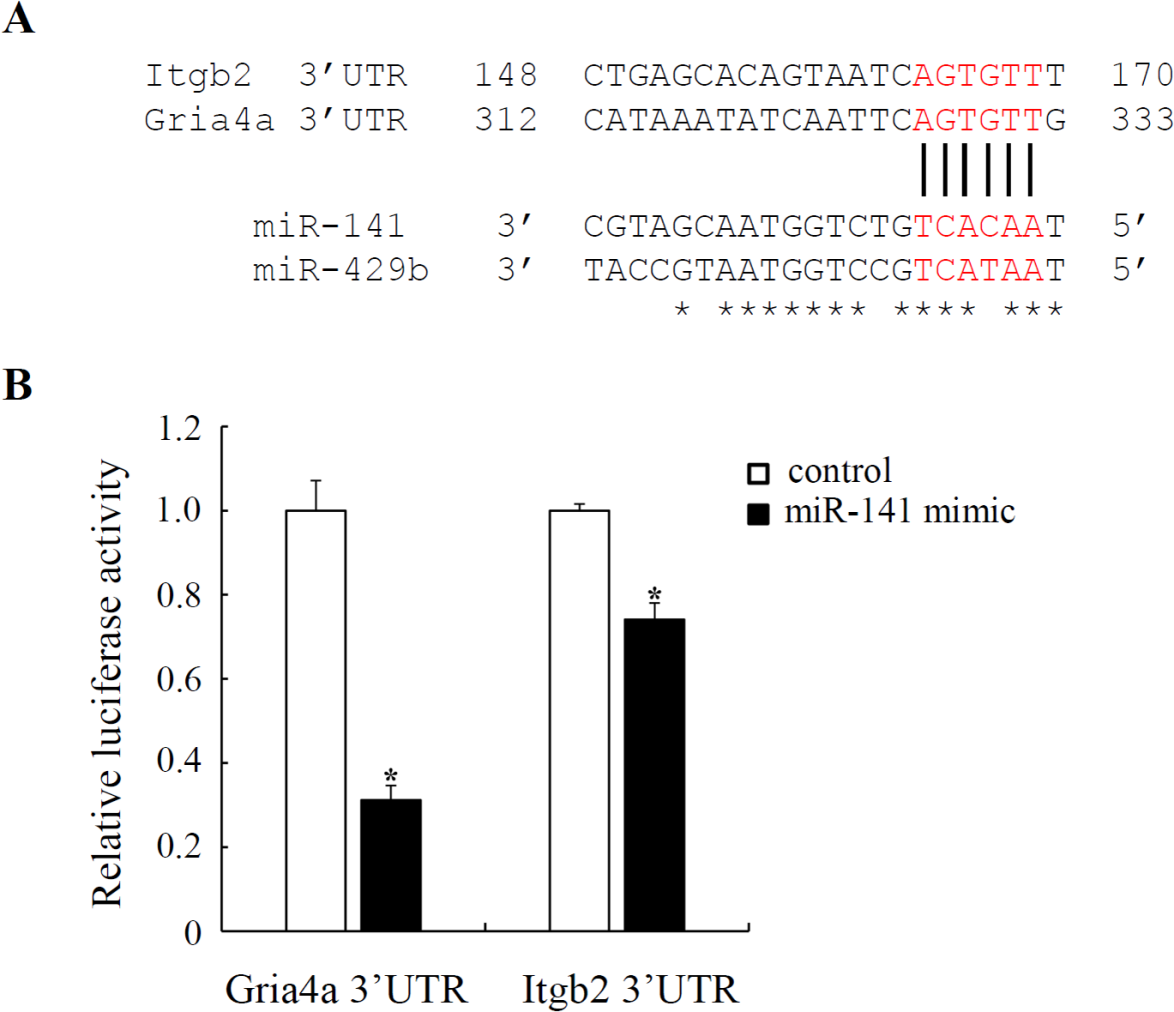
*Itgb2* and *Gria4a* are targets of miR-141. (A) Sequence alignment of *Itgb2* and *Gria4a* 3’UTR to miR-141/-429b. Red color letters represent seed sequences of miR-141/-429b and their binding positions. (B) The activities of pmirGLO reporters that is linked to the 3’UTR of *Itgb2* and *Gria4a* were suppressed by miR-141 when compared to its control oligonucleotide. *p<0.05 indicates the significant difference.

To determine whether there are direct interactions between miR-141-3p and PI3K-AKT or GPCR signaling pathway, we used dual-luciferase reporter assays to measure the inhibitory effect of this miRNA on Itgb2 and Gria4a. There is one binding site for miR-141-3p in either Itgb2 or Gria4a 3’UTR (Fig 8A). We sub-cloned the 3’UTR of Itgb2 or Gria4a into the pmirGLO vector, and co-transfected each construct with miR-141 mimic or its appropriate control into HEK293 cells. The results showed that miR-141-3p down-regulated luciferase activity by 69% (±3%) in Gria4a 3’UTR and 26% (±4%) in Itgb2 3’UTR, respectively (Fig 8B). These results strongly support the prediction of Itgb2 or Gria4a as direct targets of miR-141-3p.

## Discussion

In vertebrates with XY sex-determining system, the expression of sex-determining gene on Y chromosome leads to the development of male phenotypes and testis. Yellow catfish, an important aquaculture fish in China has XY sex-determining system and displays sexual size dimorphism as male grows 2–3 times faster than female. 454 pyrosequencing and illumina sequencing studies have been performed to compare differentially expressed genes and pathways between XX ovary and XY testis and provide a valuable genomic resource for studying fish reproduction, sex determination and differentiation [27,28]. However, there are limited studies regarding the gene expression difference between XY and YY fish. Therefore, we used solexa sequencing technology to compare mRNA expression between XY and YY testis of yellow catfish.

In fully mature 18 month-old Nile tilapia, the sperm quality and quantity of XY and YY males were the similar [22]. However, Herrera et al. found that YY tilapia has superior reproductive capacity than XY fish, since the primordial germ cells and spermatogenic cells in YY were larger than XY fish during gonad development and the lobules, blastemal of the reproductive duct and mature sperm cells appeared earlier in YY than XY fish. Moreover, YY tilapia has bigger testis, thicker somatic tissues and more spermatogenic cells, as well as matures earlier than XY fish [29]. In yellow catfish, we also found that YY matures earlier and has superior reproductive capacity than XY fish, as larger spermatogenic cyst and more spermatids were observed in YY fish [23]. Interestingly, androgen receptor has a relative higher level of expression in efferent ducts of YY testis compared to XY testis in rainbow trout [20]. All these studies suggest that YY testis has substantial difference in histology, structure and gene expression compare to XY testis.

It is important to know the context under which specific signaling pathways regulate sperm maturation as well as testis development in YY that matures earlier than XY fish. The PI3K-AKT signaling pathway plays essential roles in testis development and spermatogenesis, as loss of p110beta subunit of phosphoinositide 3-OH kinase impaired spermatogenesis and lead to defective fertility [26]. Activation of the PI3k/Akt pathway by membrane progestin receptor-alpha stimulated sperms leads to hypermotility in Atlantic croaker [25]. Lgr4, one of the orphan GPCRs regulates sperm development and fertility [30]. Testosterone signaling is mediated by a G-protein-coupled receptor and its interactors [31]. We have found more PI3K-AKT and GPCR signals in YY than XY yellow catfish, such as *syk*, *prlr* and *kiss1r*, coincides with a higher degree for testis maturity and advantageous spermatogenesis in YY than XY yellow catfish [23]. As one of the cytoskeletal component of spermatic flagella, tyrosine-phosphorylated Syk could bind and phosphorylate to its downstream part PLCγ1 and regulate the metabolic hyperactivated motilityof spermatozoa [32–34].

In mammals, increased expression of miR-141/429 was associated with defects of spermatogenesis [35–37]. High dose of 17α-ethinylestradiol (EE2) resulted in upregulation of miR-141/429 and impairment of spermatogenesis [23]. In our study, miR-141/429 was predicted to target several factors in PI3K-AKT and GPCR signaling pathways that were involved in testis development and spermatogenesis. Further characterization of the interaction of miRNAs and their targets could contribute to a better understanding about the molecular mechanisms of testis develop and spermatogenesis.

## Materials and Methods

### Solexa library construction and sequencing

All experimental procedures involved in this study were permitted by the Institutional Animal Care and Institute of Huazhong Agricultural University. The total RNAs of XY (4 individuals) and YY (3 individuals) testes were the same biological sample as described before [23]. The RNA integrity analysis was performed by the Agilent 2100 Bioanalyzer (Agilent Technologies). An amount of 3μg total RNA was used for libraries construction. All the following process was performed by using the Illumina RNA Sample Preparation Kit, following the manufacturer’s protocols. The mRNA was concentrated by oligo(dt) magnetic beads and then made into short fragments (~200bp) to work as templates for synthesizing the first strand cDNA. The double strand cDNA libraries were synthesized and purified by agarose gel purification, in which, the cDNA fragments were coupled by sequencing adptorsat the 5’ and 3’ ends. The male (XY) and super-mal (YY) libraries were sequenced on an IlluminaHiSeq 2000 platform. After removing the adaptor, low quality bases (Length threshold value>35bp), 3’-end low quality bases (Quality threshold value>20) and the reads containing the “N”, the clean reads were assembled into contigs with the Trinity software [30]. The generated contigs were clustered into unigenes by performing TGICL software system [31]. The raw reads of yellow catfish gonad have been deposited to the NCBI database (accession no: SRR1845493).

### Quantification of differential expressed unigenes

To detect the differential expression of unigenes, DESeq software package with RPKM (Reads Per Kb Million reads) method was performed to quantify the expression of two expression profiles. The formula applied was $\text{RPKM}=\frac{10^{6}C}{NL/10^{3}}$, in which C is the number of reads mapper to merged transcripts, N and L are the total mapped reads and base number of one unigene [38]. The False Discovery Rate (FDR) method was applied in multiple hypothesis of test to correct significant levels and eliminate influence of random fluctuations and errors [39]. After calibration, the ratios of RPKMs were used to calculate fold-change with threshold value cut-off of 2-fold and Negative binomial distribution hypothesis-testing with *P*< 0.05 [40].

### Functional annotation and GO/KEGG enrichment analysis

The unigenes were searched against databases of NCBI nr, SWISS-PROT, TrEMBL, Cdd, pfam and KOG by Blast X with a cut-off E-value of 10e^-5^. The results of BlastX annotation were uploaded on Blast 2 Go to generate Gene Ontology annotations and mapped to the categories of GO database ((geneontology.org/page/download-annotations)[41]. The results of BlastX were also searched against the Kyto Encyclopedia Genes and Genomes (KEGG) in Blast 2Go. All the differential expressed unigenes were mapped to KEGG database with counted numbers of involved interactive metabolic pathways (*p*<0.05). To investigate which GO item and signaling pathways the DEGs participated in, all of the clustered DEGs were mapped back to the GO and KEGG databases. Each enrichment item was corresponded to a specific enrichment score that was calculated by the formula, score value = mnMN. The statistical significance of the GO enrichment was evaluated by the hypergeometric distribution testing, P = 1-$\sum_{i=0}^{m-1}\frac{\left(\begin{array}{c} M\\ i \end{array})(\begin{array}{c} N-M\\ n-i \end{array}\right)}{\left(\begin{array}{c} N\\ n \end{array}\right)}$, where N is the number of unigenes with GO annotation, n is the number of DEGs with GO annotation, M is the number of unigenes with one specific GO annotation and m is the number of differently expressed unigene with one specific GO annotation[42].

### Quantitative real-time PCR (qRT-PCR)

Briefly, 1μg of total RNA was reverse transcribed by using PrimeScriptRT reagent Kit (Takara) according to the protocol. The qRT-PCR reaction was performed in a 20μl reaction volume using the Roche LightCycler 480 with SYBR Green PCR master mix(Roche) and three biological replicates were conducted for each reaction. The β-actin was selected as an internal reference to normalize the Ct values of each reaction by using the 2^-ΔΔCt^ method [43]. The ANOVA analysis was used to perform differential expression analysis.

### Identification of direct miRNA targets and validation by dual-luciferase reporter assays

In our recent study, we found that miR-141-3p and miR-429b-3p have higher expression level in XY than YY testis [23]. To investigate potential targets of miR-141-3p and miR-429b-3p, first the Open Reading Frame (ORF) and 3’UTR of YY highly expressed unigenes were predicted, by searching against the vertebrate genomic database in GENSCAN (http://genes.mit.edu/GENSCAN.html). Perl scripts of both Target Scan and miRanda were performed for searching the putative targets with default parameters, including context score percentile ≥100 for Target Scanand Max_Energy≤ −20 and for miRandabased on hybrid energy and stability [44,45]

To characterize the interaction between miR-141-3p/-429b-3p and their predicted target genes, the 3’-UTR of selected putative target genes (Itgb2 and Gria4a) were inserted into the pmirGLO expression vector (Promega, USA). Hek-293T cells were seeded in 96-well plates and co-transfected with the constructed vectors and microRNA mimics or its control oligonucleotide using DharmaFECT transfection reagent (Dharmacon). 36h post transfection, the dual-luciferase reporter assay system was used to detect reporter (Firefly and Renilla) activity as described [46]. The profile of relative luciferase activities were normalized to Renilla luciferase activities.

## Supporting Information

S1 Fig Length distribution of the de novo assembled unignes. (TIF)

S1 Table The annotation of unigenes in the transcriptomes. (XLSX)

S2 Table The GO annotation of differently expressed unigenes. (XLSX)
