# Supplementary figures and images for: Comparative Transcriptome Analysis of Differentially Expressed Genes and Signaling Pathways between XY and YY Testis in Yellow Catfish
Source: PLoS One. 2015 Aug 4;10(8):e0134626. doi: 10.1371/journal.pone.0134626 (PMC4524600; doi:10.1371/journal.pone.0134626)

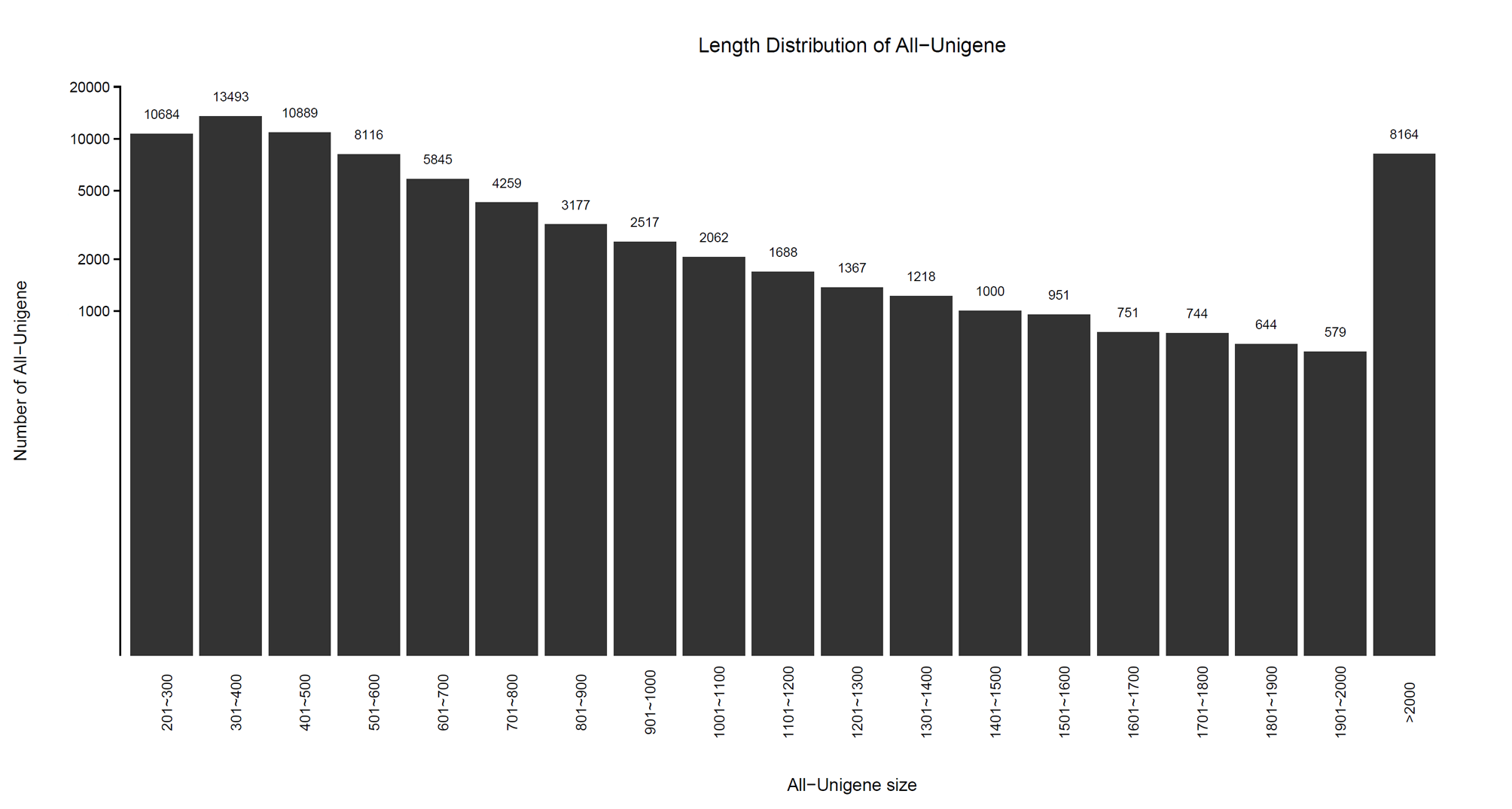

Supplement: S1 Fig — (TIF) [file pone.0134626.s001.tif]
